# Arterial Hypertension and Interleukins: Potential Therapeutic Target or Future Diagnostic Marker?

**DOI:** 10.1155/2019/3159283

**Published:** 2019-05-02

**Authors:** Daniela Maria Tanase, Evelina Maria Gosav, Smaranda Radu, Anca Ouatu, Ciprian Rezus, Manuela Ciocoiu, Claudia Florida Costea, Mariana Floria

**Affiliations:** ^1^Department of Internal Medicine, “Grigore T. Popa” University of Medicine and Pharmacy, Iasi, 700111, Romania; ^2^Internal Medicine Clinic, Iasi “Sf. Spiridon” County Clinical Emergency Hospital, Iasi, Romania; ^3^Department of Cardiology, “Prof. Dr. George I.M. Georgescu” Institute of Cardiovascular Diseases, Iasi, 700503, Romania; ^4^Department of Cardiology, “Grigore T. Popa” University of Medicine and Pharmacy, Iasi, 700111, Romania; ^5^Department of Pathophysiology, Faculty of Medicine, “Grigore T. Popa” University of Medicine and Pharmacy, Iasi, 700111, Romania; ^6^Department of Ophthalmology, Faculty of Medicine, “Grigore T. Popa” University of Medicine and Pharmacy, Iasi, 700111, Romania

## Abstract

Hypertension as a multifactorial pathology is one of the most important cardiovascular risk factors, affecting up to 30-40% of the general population. Complex immune responses are involved in the inflammatory mechanism of hypertension, with evidence pointing to increased inflammatory mediators even in prehypertensive patients. Increased vascular permeability, thrombogenesis, and fibrosis, effects that are associated with sustained hypertension, could be attributed to chronic inflammation. Chronic inflammation triggers endothelial dysfunction via increased production of ROS through proinflammatory cytokines. Increased serum level of proinflammatory cytokines such as IL-1*β*, IL-6, IL-8, IL-17, IL-23, TGF*β*, and TNF*α* in hypertensive patients has been associated with either increased blood pressure values and/or end-organ damage. Moreover, some cytokines (i.e., IL-6) seem to determine a hypertensive response to angiotensin II, regardless of blood pressure values. Understanding hypertension as an inflammatory-based pathology gives way to new therapeutic targets. As such, conventional cardiovascular drugs (statins, calcium channels blockers, and ACEIs/ARBs) have shown additional anti-inflammatory effects that could be linked to their blood pressure lowering properties. Moreover, anti-inflammatory drugs (mycophenolate mofetil) have been shown to decrease blood pressure in hypertensive patients or prevent its development in normotensive individuals. Further research is needed to evaluate whether drugs targeting hypertensive-linked proinflammatory cytokines, such as monoclonal antibodies, could become a new therapeutic option in treating arterial hypertension.

## 1. Introduction

According to WHO, cardiovascular diseases (CVDs) add disability-adjusted life years and, in 2015, caused 17.7 million deaths [1]. Arterial hypertension (HTN) is a major CVDs risk factor and multifactorial disease, affecting 30-40% of the population and causing 7.5 million deaths worldwide [2]. Despite numerous (non)pharmacological measures to prevent it/slow it down, HTN prompts 62% of strokes and 38% of heart diseases in developing countries [3]. Increasing evidence reveals HTN as a chronic inflammatory state [4, 5]. Whether inflammation contributes to HTN or HTN generates systemic inflammation remains to be seen. Inflammatory cytokine basal levels (IL-1*β*, IL-6, IL-8, IL-17, IL-23, TGF*β*, and TNF*α*) are higher in hypertensive patients. These cytokines (IL-6) trigger hypertensive responses to angiotensin II infusion even in normotensive individuals, which presents new potential therapeutic targets [6–8].

## 2. Inflammation in Arterial Hypertension

A key component in the pathophysiology of HTN is inflammation [9–11]. Not only does it determine HTN development and/or progression, but it also leads to end-organ damage. Metabolic/chemical, mechanical (wall stretch), or infectious endothelial aggressions trigger complex immune reactions, leading to a proinflammatory state [12, 13]. Inflammation, in turn, promotes endothelial dysfunction and atherosclerosis through reactive oxygen species (ROS), a downstream product of cellular and soluble immune factors [14–16]. Consequently, ROS stimulates proinflammatory cytokine secretion, increasing IL-6 expression and decreasing NO availability [8]. Studies have shown that inhibition of these ROS led to blood pressure reduction through endothelial function improvement via increased nitric oxide (NO) production [8, 9, 16].

### 2.1. RAS and Proinflammatory Cytokines

The implication of renin-angiotensin-angiotensinogen system (RAS) in the pathogenesis of HTN has been long known.

Interestingly, several immune cells (T lymphocytes, dendritic cells, and macrophages) express angiotensin 1 receptors (AT1R). By binding to AT1R, angiotensin II determines immune cells differentiation and subsequent proinflammatory cytokine production, such as IL-6, IFN-*γ*, and TNF*α* [5]. In addition, by acting on P-selectins and adhesion molecules, it increases leukocytes adhesion and migration. Moreover, angiotensin II impacts the immune system even in the absence of vasoconstrictor effects. This may explain the role of RAAS in HTN pathogenesis as an inflammatory disease [17]. In fact, it seems that angiotensin II contributes not only to HTN development, but also to HTN-mediated organ damage. In turn, proinflammatory cytokines, such as TNF-*α*, determine increased angiotensin converting enzyme (ACE) production, which contributes to inflammatory-mediated HTN [11, 15, 16].

Another pathway through which angiotensin II contributes to inflammation is by stimulating NADH and NADPH oxidase, determining increased reactive oxygen species (ROS), including superoxide production. Moreover, IL-6, stimulated by angiotensin II, in turn determines increased NADH and NADPH production, altering vascular permeability, constriction, and fibrosis degree [18–20].

### 2.2. ROS Regulation via Inflammation

It is known from preclinical models that the imbalance between reactive oxygen species production and degradation is involved in the HTN mechanisms. Because excessive ROS enhances cellular processes like differentiation and apoptosis and controls vascular tone and endothelial function, it contributes to endothelial dysfunction [21]. Increased ROS generation and reduced antioxidants levels (nitric oxide) lead to oxidative stress. ROS production involves both cellular and mitochondrial levels, with the latter being the main endogenous source. ROS molecules including xanthine oxidoreductase, uncoupled NO synthase (NOS), nicotinamide adenine dinucleotide phosphate (NADPH) nitric xanthine oxidase (NOX), and mitochondrial respiratory enzymes play a role in the HTN development [21, 22]. The production of mitochondrial ROS depends on the activation of the mitochondria ATP-sensitive potassium channels (mKATP), opening of mitochondrial permeability transition pore (mPTP), and the pH gradient in the inner membrane. Through mitochondrial ATP synthesis function, mitochondrial respiratory chain O_2-_^∙^ is converted by SOD2 (superoxide dismutase) to H2O2, which is a neutral molecule. Authors have shown that mitochondrial ROS enhances NADPH production and vice versa. Therefore, mitochondrial ROS generation, as a continuous feed-forward cycle, is redox-dependent.

Both end-organ dysfunction and HTN could be a result of the mitochondrial oxidative stress, which is the outcome of overproduction of mitochondrial superoxide and reduced SOD2 function. Interchanges between mitochondrial oxidases and NOXs are highly implicated in cellular ROS production [23].

Endothelial and plasma xanthine oxidase (XO) have been linked to HTN induced atherosclerosis and associated end-organ-damage [24]. Increased oxidative stress enhances RAAS activation in hypertensive subjects. This leads to prolonged redox signaling and reduced NO bioavailability in renal microvasculature [25]. Emerging evidence proposed that inflammatory cytokines such as IL-6 influence eNOS activity and expression, NADPH oxidase with subsequent impact on NO and superoxide levels [11, 22, 26]. Studies have shown that accessory proteins such as p22phox, p47phox, p67phox and Rac1 and NOX (the main catalytic of NADPH oxidase) stimulate oxidase activity and superoxide generation. Besides NADPH oxidase, other enzymatic endothelial superoxide sources are cytochrome P450, uncoupled eNOS, cyclooxygenase, and xanthine oxidase [27].

The proinflammatory state of hypertensive patients is supported by many studies that revealed increased serum levels of C-reactive protein [16, 28–31]. Moreover, these patients had histological arterial wall inflammation attributed to proinflammatory cytokines secretion [15, 16], insulin resistance, and increased atherogenic lipoprotein levels [29–31].

### 2.3. Endothelial Dysfunction in Hypertensive Patients

The innermost layer of the blood vessels, the endothelium, was first considered a passive barrier between blood and vascular wall. Bunting et al. demonstrated in 1976 [29] that the arterial wall can synthetize both endothelium-derived relaxing factors (EDRF) like NO, prostacyclin (PGI_2_), and hydrogen sulfide (H2S) and constrictor factors including angiotensin II (Ang II) and endothelin-1 (ET-1) [30].

**Figure 1:**
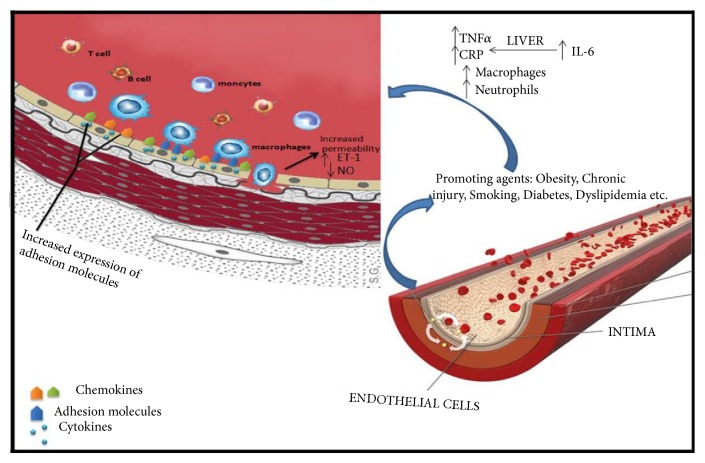
Etiology of the inflammatory process. Low level chronic inflammation increases the concentrations of markers and of inflammatory cells, leading to increased production of C-reactive protein (CRP) by the liver, in response to interleukin-6 (IL-6), which provokes a reduction in vasodilation and an increase in vascular damage. TNF-a: tumor necrosis factor alpha; IL-6: interleukin-6; CRP: C-reactive protein; NO: nitric oxide; ET-1: endothelin-1.

Cardiovascular risk factors such as dyslipidaemia, obesity, and diabetes, through their inflammatory state, promote endothelial dysfunction. Mast cells, T lymphocytes, dendritic cells, activated neutrophils, and platelets interact to produce an inflammatory response, with increased production of proinflammatory cytokines, ROS, and adhesion molecules [14, 31–33]. In some autoimmune diseases such as psoriasis or rheumatoid arthritis [34, 35] anti-endothelial antibodies cause endothelial cells to release adhesion molecule and chemokines. Elevated serum levels of IL-6 lead to increased hepatic inflammatory markers production such as CRP [17] and increase vascular permeability, cell apoptosis, and thrombosis [36] (Figure 1). Moreover, the link between endothelial dysfunction and inflammatory cytokine production has been emphasized by studies that attribute increased IL-6 production to mechanical endothelial stretch in hypertensive individuals, with a subsequent decrease in NO production [8].

Thus endothelial dysfunction is an early predictor of atherosclerosis and cardiovascular events and mortality [37], as patients with coronary artery disease [18], peripheral arterial disease [38], or heart failure [39] have shown endothelial dysfunction. Ridker et al. have pointed out to the association between increased serum levels of IL-6 and the risk of myocardial infarction [40]. Increasing evidence shows the importance of inflammation and oxidative stress in cerebral and cardiac microvascular dysfunction as well as the specific role of B-lymphocytes in cytokine production [41].

### 2.4. Role of Interleukins in Inflammation and HTN Development

Cytokines are involved in several cellular processes, ranging from inflammation to both tissue damage and regeneration. They induce immune cells recruitment and activation and play an important role in arthrosclerosis development [42, 43]. Proinflammatory stimuli determine mast cells cytokine production, triggering endothelial expression of adhesion molecule such as vascular cell adhesion protein 1 (VCAM-1), P- selectin, and plated activating factor (PAF) [19]. Leukocyte recruitment and adhesion promote vascular and extracellular matrix remodeling through increased fibrosis and hypertrophy with a subsequent reduction in vascular lumen diameter [44, 45].

Apart from their effect on blood pressure, interleukins, as key mediators of inflammation, seem to play a role in end-organ damage in hypertensive individuals [19, 43, 44]. IL-6 has shown profibrotic effects, determining both vascular and myocardial fibrosis [46]. In relation to increased arterial stiffness in the context of inflammation, cardiac hypertrophy can be explained as a response to immune cell- induced mechanical changes [47, 48].

**Figure 2:**
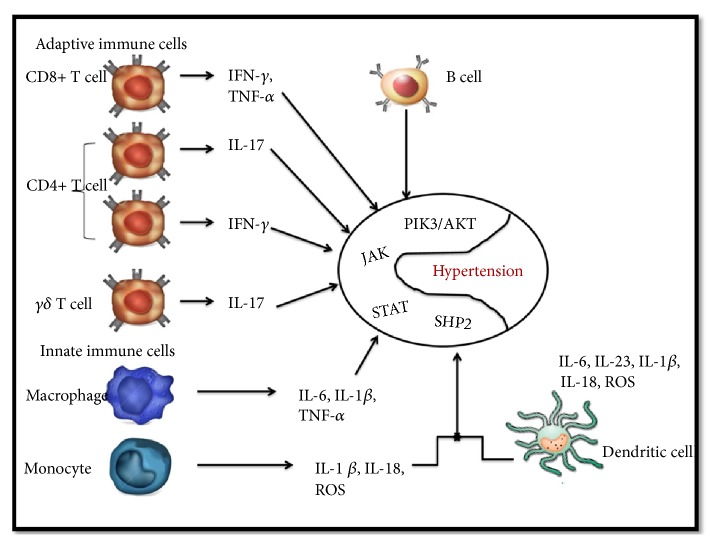
The inflammatory-mediated HTN process, immune cells via different signaling pathways. The known routes, like signal transducer and activator of transcription -1, -3, and -5 (STAT), janus-associated kinases (JAK), domain containing phosphatase (SHP2) or extracellular signal related kinase and phosphatidylinositol-3-kinase (PIK3/AKT), depend on the specific interleukin production.

Both immune and endothelial cells contribute through their interaction to the inflammatory state in hypertensive patients. The balance between proinflammatory (IL-1*β*, IL-6 IL-12, IL-18, IL-17, or IL-23) and anti-inflammatory cytokines (IL-4, IL-10) is tightly regulated and directly involved in CVDs, not only in HTN development per se, but also in mediating hypertensive end-organ damage such as ventricular remodeling and renal and cerebral involvement [20, 49]. In this inflammatory-mediated HTN process, adaptive or innate immune cells produce proinflammatory cytokines through different signaling pathways (Figure 2).

For example, IL-6R (interleukin-6 receptor) is stimulated in HTN inflammation, using STAT signaling via gp130 subunit and specific isoforms which enhance NADPH oxidase and eNOS with reduced oxide nitric levels, and increases in vascular superoxide. These actions lead to raising vascular permeability, immune cell recruitment, endothelial activation, and dysfunction. Interestingly stimulated soluble IL-6R, which reacts via membrane-anchored metalloproteinase ADAM10/ADAM17 signaling, leads to endothelial production of monocyte chemoattractant protein-1 (MCP-1). Moreover, other cytokines like IL-17 which are mainly produced by CD4 T cells or IFN-*γ* exert their inflammatory effect via similar pathways [50].

### 2.5. IL-1 and Hypertension

IL-1 is considered to be an “early-response” cytokine, involved in energy homeostasis and inflammation, connected to metabolism mechanisms [51]. Recent observations linked elevated levels of CRP as an indirect marker of IL-1 activity in the context of low-grade inflammation to HTN development [52]. IL-1 pathway seems to play an important role in atherosclerosis, with IL-1*α* and/or *β* promoting the expression of VCAM-1, ICAM-1, and E-selectin [53], with increased endothelial cell permeability, adhesion molecules expression [54]. Furthermore, endothelin-mediated vasoconstriction seems to be increased by IL-1 and TNF*α* [55]. IL-1*β*, produced by macrophages and monocytes [56], promotes the release of IL-6 and its downstream cytokine, IL-17a, while IL-18, also a member of IL-1 family, stimulates the production of IL-2, IL-12, and IFN-*γ* [57, 58]. In hypertensive patients, the peripheral blood monocytes (PBMCs) are preactivated with an increased release of IL-1*β* and tumor necrosis factor (TNF) [59]. In chronic hypertensive patients with/without end-organ damage, like vascular/myocardial remodeling and renal dysfunction, whether the levels of IL-1*β* and IL-18 are the cause or the effect of the disease remains to be seen [60]. A study conducted by Hunag et al. [61] showed that the presence of 511T allele in the promoter region of the human IL-1*β* was associated with HTN development. Moreover, several studies concluded that allele 2 of a variable number of tandem repeats (VNTR) in the intron 2 of the IL-1 receptor antagonist (IL-1 RN) gene is linked to HTN in English [62], Australian [63], and Caucasian population [64]. However, association of IL-1*β* -511C/T and IL-1 RN 86 bp VNTR polymorphisms was not relevant in the aetiology of HTN in a study conducted on 500 Pakistani Pathan subjects [65]. Also a cross-sectional study [66] conducted on 625 Japanese suggested that TT genotype of interleukin-1*β* C-31T polymorphism may have a minor role in HTN development and that this association is regulated by serum *β*-carotene levels. However, another previous study reported no association between -1B C-31T polymorphism and HTN in Caucasians [63]. Therefore, more studies are required to identify the role of cytokine gene variants and their immunologic pathways' contribution to HTN aetiology.

Studies suggested that IL-1 receptor antagonist plays a great role in metabolic effects and that one synonymous SNP (rs315952, Ser133Ser) of the IL1RN gene was associated with development of obesity and hypertension [67]. Therefore, a therapeutic option targeting several comorbidities may be reducing the activity or expression of Il-1*β* or IL-1 receptor 1 [58, 68]. Also IL-1h from the IL-1 proinflammatory superfamily has a variety of activities, including consistent effects on the atherosclerotic cell types [69, 70]. Barbieri et al. [71] showed in 537 subjects with insulin resistance syndrome that serum levels of IL-1h and IL-1ra were the only predictors of elevated diastolic blood pressure. Liu Y et al. [6] reported that endothelial cells activation leads to increased release of IL-1 h and TNF*α* in spontaneous hypertensive rats. Furthermore, interleukin-1h infusion caused dose-dependent vasopressor response with increased blood pressure [72].

### 2.6. Role of IL-6 in Inflammation and Hypertension

IL-6 is a pleiotropic cytokine, with both proinflammatory and anti-inflammatory effects [73] and multiple physiological roles. 30% of circulating IL-6 originates in adipose tissue. IL-6 promotes B cells differentiation, T cells expansion and activation, and acute-phase response regulation. Given its effects, it is now considered an important cardiovascular risk biomarker [74, 75]. Normal concentrations are relatively low (1-5 pg/ml) but are elevated in autoimmunity, infection, or cancer [25]. Signaling via gp130, the signaling subunit of the IL-6 receptor, is also needed for cell survival, growth, and function, especially Th1 and Th2 [76]. IL-6 is essential in the generation of Th17 lymphocytes via STAT3 and promotes IL-10 production, an anti-inflammatory cytokine [26, 77]. It contributes to acute-phase response by stimulating CRP hepatic synthesis, fibrinogen, and plasminogen activator inhibitor-1 (PAI-1) [78, 79], also influencing B cells proliferation [80]. Ang II, TNF-*α*, ET-1, IL-1*β* and tissue hypoxia/ischemia stimulate IL-6 secretion with increased ROS production [81, 82]. Several studies showed that angiotensin II infusion caused both blood pressure and IL-6 serum levels increase in hypertensive patients [83, 84].

IL-6 may be involved in the pathogenesis of HTN [40, 82, 85] through its effects on vascular inflammation and stiffness and endothelial dysfunction [29, 70, 86]. Moreover, it stimulates arterial wall collagen synthesis, inhibits its degradation, and stimulates fibrinogen production [11]. Interestingly, increased IL-6 levels have been found in atherosclerotic plaques [87]. IL-6 may even show promise as a biomarker; several studies have proposed increased IL-6 and TNF-*α* serum levels as independent risk factors for the development of high blood pressure in apparently healthy patients [88]. A correlation between plasma levels of IL-6 and TNF-*α* with coronary endothelial dysfunction was found in hypertensive patients [40, 88].

Its effects on cardiac contractility reduction [89] and LV (left ventricular) remodeling- fibrosis, hypertrophy and dilatation [90, 91], support the association between IL-6 and development of end-organ damage in hypertensive patients. Janssen et al. reported in an experimental model the association of cardiac fibrosis with increased levels of IL-6 [92]. Continuous IL-6 infusion in hypertensive rats was shown to determine diastolic dysfunction irrespective of blood pressure values [93].

Consequently, IL-6 inhibition in myocardial infarction patients led to a lesser degree of left ventricular fibrosis [94]. However, Lai et al. [95] found no myocardial hypertrophy regression in IL-6 knockout (KO) mice.

The relationship between IL-6, Ang II, and aldosterone could explain the role of IL-6 in the development of HTN and provide a new therapeutic target [55, 96–98]. Sterpetti et al. [98] revealed that Ang II-mediated vasoconstriction and endothelial dysfunction may be upregulated by IL-6. Studies suggest that the renal JAK2/JAK3 pathway with its IL-6-dependent activation may play a role in Ang II mediated HTN [99]. Regarding the involvement of innate immunity in the disease pathogenesis, it seems that the loss of T cells attenuated deoxycorticosterone acetate (DOCA)-salt hypertension leads to a subsequent decrease in renal IL-6, TNF-*α*, ET-1, monocyte chemoattractant protein 1 (MCP-1), and ROS [100].

The role of IL-6 in the renin-angiotensin-aldosterone system (RAAS) could also explain the renal dysfunction in hypertensive patients. Ang II enhanced IL-6 levels in the kidneys with profibrotic and ET-1 gene expression induction, suggesting a possible role in chronic kidney disease (CKD) [101]. Moreover, it seems that IL-6 has a major effect in mediating the rightward shift in the renal pressure-natriuresis relationship [83] and that Ang II dependent HTN is associated with renal IL-6/ET-1 axis [101]. Conversely, IL-6 infusion in pregnant mice enhanced angiotensinogen expression in cultured renal proximal tubular cells [102], altering renal function [103, 104]. Interestingly, in haemodialysis patients, reduced levels of albumin are associated with IL-6 concentrations and increased mortality [102].

IL-6 also plays an important role in pulmonary hypertension and fibrosis [103, 105, 106]. The association between IL-6 and hypoxia is supported by increased IL-6 mRNA pulmonary levels in hypoxia-exposed mice and the partial protection against chronic hypoxia in IL-6 KO mice [106].

Prenatal IL-6 exposure has also shown to dysregulate hypothalamic-pituitary-adrenal (HPA) axis during adulthood [107]. Chronic uterine perfusion pressure reduction in (RUPP) in pregnant rats determines an increase in arterial pressure mediated by IL-6 [104, 108]. Moreover, elevated levels of both TNF*α* and IL-6 were identified in women with preeclampsia [109].

IL-6 gene deletion failed to affect Ang II salt induced HTN development in mice, but prevented renal dysfunction and myocardial remodeling and inflammation [110]. However, another study associated IL-6 deletion with adverse cardiac remodeling and fibrosis [111].

G to G mutations near the transcriptional start region of IL-6 (rs1800795) increase its serum concentrations and are associated with coronary artery disease (CAD) development [112, 113]. IL-6 promoter region polymorphism seems to be linked to HTN in Japanese women [114]. Single nucleotide polymorphisms (SNPs) in the -174 and -572 positions are associated with ischemic stroke [115] and CAD [116]. R Pola et al. proposed that the 174 G/C polymorphism does not affect HTN susceptibility in an elderly Italian population [117]. However, there seems to be an association between 174G/C polymorphism and left ventricular hypertrophy in dialysis hypertensive patients [118].

Several studies, including the PEGAS study [119, 120], demonstrated that the presence of 174 G/C or CRP 1846 G/A polymorphisms in specific populations increased the risk of stroke after cardiac surgery. These results suggest that ethnicity and gender might enhance the phenotypic expression of IL gene polymorphism.

IL6 _572C4G polymorphism (rs1800796) influences fibrinogen and CRP levels with no association with HTN [121]. Moreover, IL6 _572G4C polymorphism was associated with lower IL-6 levels after coronary artery bypass grafting surgery (CABG) [122]. He Ma et al. demonstrated that IL6572 C>G polymorphism correlates with HTN development [123]. A genetic variant of this polymorphism found in southern Chinese patients is rs8192284 on the IL6 receptor gene (IL6R) which appears to be associated with lower IL-6 concentrations [124]. The C alleles of rs1800795, rs1800795-, and rs1800795 were all associated with increased CAD risk [125]. Furthermore, in a Chinese population, the 634C/G polymorphism was associated with atrial fibrillation (AF) and HTN [126]. Considering these findings, further studies are required in order to fully understand the implications of IL-6 in the pathogenesis of HTN and its end-organ damage and the possible development of new antihypertensive drugs targeting inflammatory cytokines [127].

### 2.7. IL-17, Il-23, and IL-17/23 Axis in Hypertension Mechanisms

IL-23 is stimulated by antigen-stimulated macrophages and dendritic cells. It enhances T helper cells development (Tn17/ThIL-17) which secrete IL-17. IL-23 acts also in an autocrine/paracrine manner by promoting the release of other proinflammatory cytokines like IL-1, IL-6, and TNF-*α*.

The IL-17 family is formed by 6 members ranging from IL-17A to IL-17F. Despite several monoclonal antibodies targeting IL-17A, IL-17F, or the IL-17RA being used as autoimmune disease treatment (i.e., psoriasis), their effect on blood pressure has not been established [128].

There are 3 types of Th cells: Th1 cells which produce interferon-g (IFN-g), Th2 cells which produce IL-4 and IL-5; and IL-13, a third type of Th cells that secrete IL-17A, IL-17F, and IL-22 [129, 130]. The latter have a controversial role in inflammation with both atherogenic and protective roles being observed [131].

IL-17- and IL-23-producing cells have been shown to be involved in the pathogenesis of atherosclerosis [12, 131].

IL-17, by stimulating proinflammatory cytokine production, fibroblast proliferation, and profibrotic gene expression, plays a role in remodeling after post-myocardial infarction [132]. Moreover, IL-17 also interferes with tissue damage, inflammatory vascular disease [133, 134], and CAD [135]. In turn, IL-17 induces IL-1*β*, IL-6, and TNF-*α* secretion, also associated with endothelial dysfunction and hypertension [11, 24]. To support these findings, IL-17A inhibition normalized inflammatory aortic wall infiltrate in mice [136]. IL-17 synthesis in the aortic media is also stimulated by Ang II infusion in both mice and humans [136]. Additionally, Zahra et al. [137] reported increased IL-17 levels in chronic heart failure (CHF) patients. Studies revealed that IL-17 and Ang II correlated with refractory HTN in haemodialysis patients [138, 139]. Interestingly, IL-1 is essential for Th17 development, and whether IL-1 and IL-23 act at the same molecular level remains to be seen. Moreover, IL-17/IL-23 axis deficiency in mice quickens DOCA Ang II-induced albuminuria in hypertensive kidney injury [140].

Since IL-17A is linked to Ang II induced HTN, renal dysfunction, and renal sodium transporter modulators, studies suggest that antibodies directed against IL-17A or the IL-17RA receptor subunit may be a novel adjunct hypertension therapy [141].

### 2.8. Other Proinflammatory Interleukins

There are more cytokines involved in the inflammatory mechanisms of HTN.

Buemia et al. showed that in patients with essential hypertension, IL-8 and ICAM-1 significantly increased the Ca2+ dependent K+ outflow in red blood cells [142]. IL-12, mainly secreted by macrophages, is a inducer of Th-1 type cellular immune response [143].

Moreover, IL12B ^*∗*^A/^*∗*^A genotype was less frequent in patients with a history of stroke and IL12B ^*∗*^A/^*∗*^C genotype had increased risk of stroke [120].

Authors thought IL-5, with its anti-inflammatory and prothrombotic cytokine, mediated atherogenesis [144]. Kaibe et al. showed that Il-15 serum concentration were higher in hypertensive patients with severe organ damage [145].

HTN, through its effects on the vascular wall, leads to increased macrophages and mature IL-18 cells production. The subsequent natural killer and T cell maturation together with IL-2 secretion determines IL-4, IL-10, IL-13, and IFN- *γ* release [146].

Also, IL-18 stimulates ROS production from mechanical stress mediated NADPH oxidase and accentuates superoxide-induced HTN, suggesting a role in Ang II induced hypertension [147]. According to Jussi et al., IL-18 gene promoter region 2137G/C polymorphism modulates HTN effect on CAD and coronary atherosclerosis development [148]. IL-18 is associated with coronary stenosis and acute fatal coronary syndromes, leading to increased interferon-g (IFN-g) and matrix metalloproteinase (MMPs) secretion, which are linked to atherosclerotic plaques instability [149].

In PRME study, higher IL-18 levels seem to predict future coronary events in healthy men irrespective of blood pressure values [150]. However, more studies are needed to assess IL-8 reliability in predicting cardiovascular events.

IL-22 is another member related to IL-10 superfamily, with both pro- and anti-inflammatory properties [151]. The CC genotype of IL-22 gene polymorphism may be an independent risk factor in hypertensive patients with CAD [152], while GG genotype had a protective effect. IL-22 (rs1179251) polymorphism in the chromosome 12 (12q15, intron 4) may be associated with HTN and/or CAD [153].

A more recently discovered member of IL-1 family is IL-33. If Rana et al. suggested that IL-33/ST2 signaling may have a possible protective role in heart failure, no studies have investigated IL-33 in HTN [154].

### 2.9. Anti-Inflammatory Cytokines (IL-4, IL10) and Hypertension

IL-10 is known to be the main anti-inflammatory cytokine, produced by Th1, Th2, Th17, and epithelial cells and keratinocytes. It binds to its receptor, activating the IL-10/JAK1/STAT3 cascade. Hence, STAT3 phosphorylation is crucial for IL-10 pathway [155]. Its anti-inflammatory effects consist of suppressing IL-1, IL-6, IL-12 and TNF*α*, HLA class II, and adhesion molecules. It upregulates a tissue inhibitor of MMP-1 expression, leading to atherosclerotic plaque stabilization [156].

IL-10 could prevent aortic endothelial dysfunction caused by TNF*α* in mice [157]. In a another experimental transplant model, IL-10 deficiency was shown to accelerate atherosclerosis development and increase allograft refection in mice [158]. Also, IL-10 deficiency exacerbates Ang II induced vascular dysfunction [159]. Allograft survival in these models was prolonged after treatment with either rIL-10 or rIL-4 [160].

IL10 gene -627 C/A polymorphism was associated with HTN in a Tatar ethnic group, with the ^*∗*^C/^*∗*^C genotype preventing HTN development [120].

Another pleiotropic cytokine secreted by T and B cells is IL-4. It can also be detected at the feto-maternal interface. It inhibits inflammatory cytokine production, increases MHC class II and CD23, and promotes immunoglobulin E and G1 production [160]. Piyali et al. showed that IL-4/IL-10 cotreatment during gestation in mice with preeclampsia normalized blood pressure and endothelial function and decreased the IL-6, IFN*γ*, TNF*α*, and TGF*β* levels [161].

Cytokines play an essential role in the inflammatory processes. As their role in the development or progression of cardiovascular diseases such as HTN or atherosclerosis (Table 1) continues to be researched, new possible therapeutic paths concerning these interleukins may be discovered.

## 3. Inflammation as a Potential Therapeutic Target in Arterial Hypertension

There has been consistent evidence over the past years that inflammation is a key element in the pathophysiology of hypertension, leads to, progression, and development of end-organ damage. While hypertensive patients have been shown to present with increased levels of proinflammatory cytokines such as IL-1*β*, IL-6, IL-8, IL-17, and TNF*α*, antihypertensive treatment seems to determine a reduction in their concentration.

Considering inflammation, a new therapeutic target in hypertension is an appealing idea, especially due to its role in the pathophysiology of the disease, but more importantly, in developing end-organ damage and/through profibrotic states.

### 3.1. Cardiovascular Drugs Targeting Inflammation

Several drugs already used in cardiovascular pathologies have been shown to lower serum inflammatory cytokines level [162].

### 3.2. Statins

Statins are the most frequently used lipid-lowering agents, both in primary and secondary prevention of cardiovascular events [162–165]. Apart from their effect on serum cholesterol and nonsteroidal isoprenoids synthesis through the inhibition of 3-hydroxyl-3-methyl-glutaryl coenzyme A reductase (HMGCR), they present a range of pleiotropic effects such as plaque stabilization, antithrombotic and anti-inflammatory effects, and endothelial function enhancement [165]. There has been a debate on whether statins have blood pressure lowering effects and if these effects can be attributed to their ability to reduce serum inflammatory cytokines levels and to oppose endothelial dysfunction [162].

Statins downregulate angiotensin II type 1 (AT1R) receptors [165], increase nitric oxide production through NO synthase upregulation, and inhibit ET-1 synthesis and proinflammatory cytokines production by blocking NF-*κ*B pathway [162, 164]. They decrease high-sensitivity CRP hepatic and plasminogen activator inhibitor-1 (PAI-1) synthesis [162].

Evelien van der Meij et al. demonstrated that statin therapy has anti-inflammatory dose-dependent effects [162]. Patients treated with either simvastatin or atorvastatin showed decreased levels of proinflammatory cytokines (MCP-1, MCP-1 ligand-ICAM-1, and IL-6) in the aortic aneurysm wall as determined by quantitative real time polymerase chain reaction (QRT-PCR). IL-6 aortic wall levels correlated with MCP-1 levels. Statin therapy influenced NF*κ*B dependent inflammatory mediators such as IL-6, MCP-1, TNF*α*, and IL-1*α* and *β*. There was no difference in anti-inflammatory effects between the two statins used, and both had no effect on inflammatory cellular component.

Statin's effect on blood pressure seems to be dependent on the severity of the disease, being more pronounced with greater values of blood pressure. Some studies report a 2-5 mm Hg reduction in systolic blood pressure after statin therapy [166].

Hypertensive patients tend to have increased interleukin 1*β* secretion under Ang II stimulation compared to normotensive individuals. Simvastatin therapy reduced Il-1*β* levels independently of lipid-lowering effects [167].

Conversely, there are studies that found statins to possess no antihypertensive effect [164]. 4-week simvastatin therapy in normocholesterolemic normotensive rats had no effects on blood pressure levels, irrespective of dose and prior *β*-1 adrenergic blocker (metoprolol) infusion. However, the limitations of the study included the short period of statin administration and the use of normotensive rats, as statins' blood pressure lowering effects seem to be effective in hypertensive specimens [168].

Though their hypotensive effect is controversial when given alone, some studies [169, 170] show that statins have a synergistic effect when given with either ACEI or ARBs, suggesting that their anti-inflammatory and possible antihypertensive properties may be the result of a degree of RAAS inhibition [169].

### 3.3. Statins May Influence Hypertensive Ventricular Remodeling

Statins' effects in hypertension are not limited to blood pressure reduction. Ventricular remodeling in hypertensive rats seem to be influenced by statin therapy with a reverse-remodeling effect. Hypertensive salt-loaded rats were randomized into 4 groups: atorvastatin, losartan, atorvastatin, and peroxisome-proliferators activated receptors (PPAR-*γ*) inhibitor and saline [171]. Besides reducing blood pressure values, both atorvastatin and losartan decreased serum levels of hs-CRP and of proinflammatory cytokines IL-6, IL-1*β*, TNF*α*, and TGF-*β*. Both drugs prevented renal-damage development in hypertensive rats with losartan reducing NF-*κ*B, MCP-1, and TLR-4 levels in the kidneys. Atorvastatin had beneficial effects on ventricular hypertrophy and diastolic dysfunction. The proposed anti-inflammatory mechanism of statins included PPAR-*γ* pathway inhibition and G-protein signaling alteration through HMG-CoA inhibition.

It was suggested that statins alter vasopressor response to norepinephrine and could inhibit aldosterone secretion through cholesterol synthesis reduction, an effect that may depend on solubility [54]. Statins may have blood pressure lowering effects, but these effects are of limited potency when administered alone, being most likely synergic with other antihypertensive agents.

Some studies propose that the antihypertensive properties of statins failed to be noted in larger trials because of the smaller doses administered [162]. Moreover, many studies agree that the blood pressure lowering effects are dose-dependent [162, 163].

### 3.4. Drugs Targeting the Renin-Angiotensin-Aldosterone System

Apart from statins, first-line antihypertensives affecting the renin-angiotensin-aldosterone system (RAAS), angiotensin converting enzyme inhibitors (ACEI) and aldosterone receptor blockers (ARBs), respectively, have shown anti-inflammatory effects in addition to their blood pressure lowering effects.

Valsartan has shown anti-inflammatory effects in hypertensive patients irrespective of blood pressure reduction [54, 172]. IL-1*β* basal levels in hypertensive patients randomized to either 80 mg valsartan od or other antihypertensive drugs except ARB/ACE inhibitors showed no difference between the groups. Lipopolysaccharide (LPS) stimulated IL-1*β* secretion increased in all patients. Patients having received valsartan showed reduced LPS stimulated IL-1*β* levels. Several studies agree that hypertensive patients showed increased IL-1*β* secretion under stimulation with LPS [164, 173–176]. Treatment with losartan, amlodipine, or captopril has decreased serum IL-1*β* levels irrespective of blood pressure reduction.

IL-6, IL-8, TGF-*β*, and TNF*α* levels were determined in 286 hypertensive patients and CAD patients treated with captopril, atorvastatin, losartan, aspirin, clopidogrel, metoprolol, or nitrocontin in varying doses and combinations [176]. The results showed decreased IL-6 levels and increased TGF-*β* in patients treated with higher doses of the aforementioned drugs except for metoprolol.

There are several proposed mechanisms for ARBs and ACEIs anti-inflammatory effects. Blockage of AT1 receptors may have anti-inflammatory effects firstly by antagonizing angiotensin II and secondly by allowing AT2 receptors activation, which may enhance NO production. Angiotensin II determines stimulation of NF-*κ*B transcription factors by bonding to human neutrophils, thus determining increased cytokine production. Conversely, antagonizing Ang II may reduce inflammation in both hypertensive and normotensive patients [176, 177]. Carracso-Miguel JL et al. observed that captopril reduced IL-6, IL-1*β*, and TNF*α* serum levels in spontaneously hypertensive rats most probably due to inhibition of NF-*κ*B [175].

### 3.5. Calcium Channel Blockers

Amlodipine seems to possess the same anti-inflammatory effects as ARBs and ACEIs, decreasing serum levels of proinflammatory cytokines in hypertensive patients. Matrix metalloproteinases (MMPs) are endopeptidases secreted by myocardial fibroblasts and inflammatory cells that promote pressure-overload induced myocardial remodeling in hypertensive patients [177, 178]. IL-1*β*, angiotensin II, and ROS stimulate MMP-2 and MMP-9 secretion, which increase the extent of myocardial fibrosis and remodeling through extracellular matrix (ECM) degradation and collagen fibers production. Tissue inhibitor of matrix metalloproteinases TIMP-1 and TIMP-2 inhibits MMP-2 and MMP-9 production. The balance between the two determines the degree of ECM degradation and thus of fibrosis. Hypertensive mice treated with either amlodipine, atorvastatin, or combination of both showed decreased levels of MMP-2 and MMP-9, which is the most potent effect in the case of the combination [176]. Lercanidipine may also have additional anti-inflammatory properties, having decreased CRP, white blood cell and neutrophil counts, superoxide release, and insulin levels in hypertensive patients [179].

Table 2 summarizes the anti-inflammatory effects of cardiovascular drugs and their potential hypotensive mechanisms.

### 3.6. Immunosuppressants Effects on Blood Pressure Levels

HTN has been associated with inflammation. Prehypertensive patients also present with a low-grade inflammatory state. Given that conventional antihypertensive therapies have shown anti-inflammatory effects, it is questionable whether the ability to inhibit proinflammatory cytokines such as Il-6, IL-1*β*, TNF*α*, and IL-17 could contribute to their blood pressure lowering effects. If so, the question arises as to whether anti-inflammatory drugs could lower blood pressure in hypertensive patients, thus offering a new therapeutic option in arterial hypertension.

### 3.7. Mycophenolate Mofetil

Mycophenolate mofetil (MMF) is an immunosuppressant that inhibits inosine monophosphate dehydrogenase, thus limiting T and B-lymphocytes proliferation through guanosine depletion. Its indications include lupus nephritis and other autoimmune glomerular diseases, prevention of allograft rejection [180]. Systemic lupus erythematous (SLE) mice treated with MMF had lower blood pressure values as compared to vehicle-treated specimens. Moreover, in SLE mice receiving treatment with MMF, CD45R^+^ B cells depletion with subsequent autoantibody production inhibition was observed. These findings are connected to a previous study that linked autoimmunity with HTN development [181]. Hypertensive patients tend to have an increased level of AT1R antibodies, *α*-adrenergic receptors, and L-type voltage gated calcium channels [180–183]. Interestingly, SLE is not the only autoimmune disease in which MMF influenced blood pressure; it has proven to have similar effects in psoriasis and rheumatoid arthritis hypertensive patients [182]. Moreover, MMF tends to ameliorate renal damage in hypertensive SLE patients with a reduction in albuminuria and renal lymphocytes recruitment. Therefore, it can be assumed that MMF alleviates hypertension in SLE patients firstly through the systemic anti-inflammatory effects and secondly through the ability to prevent/ameliorate renal damage. By inhibiting glomerular immune complex deposition, MMF prevents lymphocytes infiltrates and subsequent renal inflammation that supports and even may determine hypertension development. This is in accordance with other studies [183] that confirm the blood pressure lowering effects of MMF through the reduction of renal sodium transporter.

In addition to MMF, several studies indicate the antihypertensive benefits of proinflammatory cytokines inhibition. For example, IL-17R inhibition in hypertensive mice decreases fibrosis and improves cardiac function [182]. González et al. [183] showed that IL-6 hypertensive knockout mice had preserved cardiac function as determined by left ventricle ejection fraction and a lesser degree of myocardial fibrosis and inflammation. Despite not altering angiotensin II-blood pressure response, IL-6 deletion seems to prevent cardiac remodeling and subsequent dysfunction in hypertensive mice. In addition, IL-6 knockout mice showed reduced levels of albuminuria, emphasizing the role of the proinflammatory cytokine in developing end-organ damage in hypertensive patients.

### 3.8. Monoclonal Antibodies

In spite of the pathophysiological link between HTN and certain proinflammatory cytokines, the antihypertensive effects of monoclonal antibodies remain controversial [184]. Studies have shown that IL-6 knockout mice present with a lower tendency to develop HTN when stimulated with angiotensin II as compared with control, thus making IL-6 a potential therapeutic target. Moreover, IL-6 inhibition in hypertensive specimens has been linked to decreased myocardial fibrosis and inflammation [183].

Monoclonal antibodies directed against IL-6 receptor such as tocilizumab are approved for the treatment of rheumatoid arthritis. Side effects include infections in the context of neutropenia and hepatotoxicity [185]. Despite the lack of studies regarding the effects of tocilizumab treatment on blood pressure, Hernández-Sánchez et al. are currently evaluating tocilizumab in pulmonary arterial hypertension patients [185].

Other available monoclonal antibodies directed against proinflammatory cytokines include sirukumab and sarilumab, also IL-6R antibodies, currently in phase 3 trials.

Taking into consideration its role in hypertension pathophysiology, IL-17 is a potential therapeutic target. Being stimulated by IL-6, IL-17 has been associated with HTN and AT1R autoantibodies production during pregnancy. IL-17 suppression through IL-17 recombinant receptor infusion seems to decrease blood pressure and AT1R autoantibodies during pregnancy [186]. Secukinumab is an anti-IL-17A antibody approved for psoriasis treatment [184]. However, one of its cited adverse effects is HTN.

So far, there is no monoclonal antibody approved for HTN treatment, especially because their blood pressure lowering effect is not well established. Moreover, their use as anti-hypertensive drugs is precluded by their side effects, including leukopenia, neutropenia, infections, and neoplasia development. There is a need for the development of new, safer monoclonal antibodies that could be further tested as potent antihypertensive drugs.

## 4. Discussions

Given the increasing prevalence of HTN and its effects on (cardiovascular) mortality and comorbidity, there is a constant focus on better understanding of its pathogenesis [40]. It has been known that inflammation plays an essential role in HTN [16]. Chronic inflammation enhances endothelial and tissue cells functions by promoting proinflammatory cytokine synthesis: IL-1*β*, IL-6, IL-8, IL-17, IL-23, TGF*β*, and TNF*α* [28]. These cytokines contribute to elevated blood pressure with structural and myogenic modifications via elevated levels of CRP, VCAM-1, NO, EDRF, PGI2, H2S, Ang II, and ET-1 release [19, 29, 30]. IL-1*β* in essential hypertension enhances VCAM-1, ICAM-1, and E-selectin expression with atherosclerotic effects [53, 54]. Different ethnic studies revealed that specific plasma levels of IL-1*β* polymorphisms are associated with higher blood pressure [60–64].

IL-6 could be an important cardiovascular risk biomarker [93, 102]. Likewise, various IL-6 polymorphisms remain correlated with higher HTN risk in different populations [114–126]. IL-17 and IL-23 family were linked to atherogenic effects, endothelial dysfunction, hypertension, CHF [11, 137], and Ang II induced HTN with subsequent renal injury [140]. Over the years, cytokines such IL-8, IL-12, IL-15, IL-18, and IL-22 were associated with inflammation and HTN [142–153]. Additionally, IL-4 and IL10 exert their anti-inflammatory effect by suppressing IL-1, IL-6, IL-12 and TNF, HLA II, and adhesion molecules [156].

Taking into consideration the increase in HTN prevalence and the complex pathogenesis mechanism, targeting inflammation as a possible antihypertensive therapy is a new option. Evidence showed that several cardiovascular drugs like statins, ACEI, ARBs, and calcium channel blockers lowered blood pressure and serum inflammatory cytokines concentrations [54, 162, 164, 167, 171–179]. Even if antihypertensive effects of monoclonal antibodies remain controversial, some experimental animal studies showed that IL-6 inhibition in hypertensive specimens decreased myocardial fibrosis and reduced albuminuria, emphasizing the role of the proinflammatory cytokine in developing end-organ damage [180].

Studies showed that cardiovascular drugs and immunosuppressant molecules like MMF reduced plasma levels of different interleukins in hypertensive subjects. Even if few monoclonal antibodies were proven to exert some effects, so far no monoclonal antibody has been approved for HTN treatment. These findings emphasize the role of cytokines in the pathogenesis of hypertension and end-organ damage, pointing out to a possible therapeutic target.

## 5. Conclusions/Future Perspectives

Treating hypertension is not limited to blood pressure control. One of the most important goals is preventing/counteracting end-organ damage. Taking into consideration that inflammation plays an important role in developing/maintaining both hypertension and its subsequent end-organ damage, hypertensive patients may benefit from immunosuppressive therapies as a new therapeutic option. Future prospective trials are required to assess the impact of monoclonal antibodies targeting specific interleukins on blood pressure values, end-organ damage, and systemic inflammation in hypertensive patients.

## Figures and Tables

**Table 1:** Cytokines, cytokine receptors, and their vascular impact. HTA-arterial hypertension, ATS-atherosclerosis, ST-stroke, IM-myocardium infarction, CHD-coronary heart disease, AF-atrial fibrillation, CH-cardiac hypertrophy, LVD-left ventricule dilatation, HTP-pulmonary hypertension, UA-unstable angina, CHF-chronic heart failure.

Interleukine	Receptor	Cell source	Cell Target	Cardiovascular Impact
IL-1*α*,*β*	Type I IL-1r, Type II IL-1r	Monocytes/macrophage, fibroblast, endothelial cells, B cells, epithelial cells including thymic epithelium.	All cells	HTA [52, 58], ATS [53, 54, 60], IL-1*β* polymorphism and HTA [61–64, 66, 67], ST [73]
IL-4	IL-4 *α*, common *γ*	Mast cells, T cells, basophils.	Endothelial cells, T cells, B cells fibroblast, NK-cells, monocytes, macrophages	Anti-inflammatory action on T cells [161]
IL-6	IL-6r, gp130	fibroblast, endothelial, Monocytes/macrophages, most epithelial cells including thymic epithelium.	Hepatocytes, macrophages, monocytes, T cells, B cells, epithelial cells	HTA [40, 82, 85, 88], ATS [106], IM [30], CHD [112, 113, 125], AF [126], CH [90], LVD [91], HTP [103, 105, 106], ST [119, 120]
IL-10	IL-10r	T cells, B cells, monocytes macrophages, keratinocytes, mast cells	T cells, B cells, NK cells, mast cells, monocytes macrophages	Anti-inflammatory action on T cells [120, 156, 157]
IL-17	IL-17r	CD4+ T cells	Endothelium, epithelium, fibroblast, macrophages	HTA [11], ATS [12, 131], IM and UA [135], CHF [137]
IL-23	IL-12Rb1/IL23R	Macrophages, other cell types	T cells	ATS [12, 131]

**Table 2:** Anti-inflammatory effects of cardiovascular drugs.

Effects on inflammatory cytokines Antihypertensive mechanisms Proposed References			
Statins	↓ IL-1*β*	NF-*κ*B inhibition AT1R downregulation HMC CoA inhibition (G protein coupled signalling inhibition) PPAR-*γ* inhibition Upregulates NO synthase	[162, 164, 166, 167, 174]
	↓ IL-6		
	↓ MCP-1		
	↓ ICAM-1		
	↓ MMP-2		
	↓ MMP-9		
	↓ hs-CRP		
	↓ PAI-1		
	↑ NO		

ARBs/ACEIs	↓ IL-1*β*	NF-*κ*B inhibition AT1R downregulation Decreased ACE synthesis	[8, 56, 164]
	↓ IL-6		
	↑ TGF-*β* (losartan)		
	↑ NO (AT2R)		

Calcium channel blockers	↓ MMP-2	Protein kinase pathway (MMP-2)	[174, 176]
	↓ MMP-9		
	↓ IL-1*β*		
	↓ IL-18		
	↓ CRP		
	↓ MCP-1		
	↓ ICAM-1		

IL: interleukin; MCP: macrophage chemotactic factor-1; ICAM-1: intercellular adhesion molecule-1; MMP: matrix metalloproteinase; NO: nitric oxide; ARBs: angiotensinogen receptor blockers; ACEIs: angiotensin converting enzyme inhibitors; NF-*κ*B: nuclear factor *κ*B; AT1R: angiotensinogen type 1 receptor; HMC CoA: hydroxy-methyl-glutaryl coenzyme A; hs-CRP: high sensitivity C reactive protein.
